# Frequency of Hypomagnesemia and Its Relationship With Severity Among Patients of Acute Ischemic Stroke Presenting to a Tertiary Care Hospital

**DOI:** 10.7759/cureus.58244

**Published:** 2024-04-14

**Authors:** Misbah Khanum, Umbreen Arshad, Irfan ullah, Hafiz Adnan Shakir

**Affiliations:** 1 Internal Medicine, Jinnah Hospital/Allama Iqbal Medical College, Lahore, PAK; 2 Medicine, Baba Bulleh Shah Hospital, Kasur, PAK

**Keywords:** serum magnesium, prevalence, pakistan, hypomagnesemia, ischemic stroke

## Abstract

Objective

The objective of this study was to assess the prevalence of hypomagnesemia and its association with the severity of acute ischemic stroke (AIS) in patients presenting at a tertiary care hospital.

Methodology

A total of 100 patients with AIS were included in the study. Demographic data, including age, gender, and severity of stroke, were collected. Serum magnesium levels were measured at admission, and the severity of stroke was classified as mild, moderate, or severe based on clinical criteria. The presence of hypomagnesemia was defined as a serum magnesium level below 1.8 mg/dL determined within 72 hours of onset of stroke. Statistical analysis was performed to assess the association between hypomagnesemia, stroke severity, age, and gender.

Results

The mean age of the patients with standard deviation was 65.45 ± 11.8 years, with the majority (38, 38%) aged 60-74 years. There were 53 (53%) male and 47 (47%) female patients. Hypomagnesemia was found in 35 (35%) patients, with an average magnesium level of 1.93 mg/dL and a standard deviation of 0.37 at admission. There was no statistically significant difference in the distribution of stroke severity (*P *= 0.779; *P *= 0.406) or hypomagnesemia (*P *= 0.287; *P *= 0.591) based on gender or age group, respectively. Stratification based on stroke severity showed that 16 (39%) patients with mild stroke, 10 (31.3%) with moderate stroke, and 9 (33.3%) with severe stroke had hypomagnesemia. The correlation between stroke severity and hypomagnesemia was weak (*r* = 0.099). Further, among hypomagnesemia patients, the majority were females aged 60-74 years.

Conclusions

This study found a weak positive relationship between the severity of AIS and the presence of hypomagnesemia. However, no statistically significant association was observed between gender or age group and stroke severity or hypomagnesemia. These findings suggest that further research is needed to understand the role of hypomagnesemia in AIS and its potential implications for patient management.

## Introduction

Stroke is a primary cause of acquired physical disability among adults, ranking second only to Alzheimer's disease in dementia cases and third in mortality rates within developing nations [1]. Beyond its physical implications, this neurological disorder exerts a substantial psychological and financial burden on both families and healthcare systems. This is so because survivors of stroke frequently experience severe disabilities, necessitating specialized care and extended rehabilitation [2]. Approximately 85% of stroke-related fatalities are more prevalent in low- and middle-income countries, where these regions bear 87% of the global burden in disability-adjusted life years (DALYs), amounting to a staggering 72 million annually [1]. Research has shown that stroke is linked to changes in the equilibrium of magnesium, which is crucial in sustaining the metabolism of neurons and glia [3].

In the human body, following potassium, magnesium (Mg^+2^) ranks as the second most abundant cation within the body’s cells [4], essential for maintaining cardiac excitation [5] and acting as an essential cofactor in more than 300 enzyme systems that control a variety of biochemical reactions in the human body [6]. Despite magnesium's widespread presence in food sources, magnesium deficiency remains prevalent globally, even in developed nations [7]. The 2015-2020 dietary guidelines for Americans highlight that most Americans do not meet the recommended daily magnesium intake [8]. Recent studies spanning the past 15 years have linked hypomagnesemia to ischemic stroke, with reports indicating its presence in 24% of patients with acute ischemic stroke (AIS) [9] and in 20%-65% of critically ill ICU cases. Magnesium deficiency often goes unnoticed compared to other electrolyte imbalances like hyponatremia, hypokalemia, and hypocalcemia. Hypomagnesemia frequently coexists with hypokalemia and can mask its symptoms. Common causes include reduced magnesium absorption in the gastrointestinal tract (GIT), nasogastric tube suction, magnesium-poor dietary formulas, and medications such as diuretics and aminoglycosides [5].

South Asia significantly contributes to the global stroke burden, displaying notable variations in the prevalence rates of stroke among countries in the region. Pakistan, being no exception, shows a notably higher incidence compared to Europe and various nations [10]. The National Health Survey of Pakistan, conducted by the Pakistan Medical Research Council, revealed an estimated annual stroke incidence of 25/100,000, translating to approximately 350,000 new cases annually [9]. Similarly, the mortality rate linked to stroke is substantial, ranging from 7% to 20%. Complications affect up to 63% of stroke patients, with as many as 89% requiring assistance for daily activities, underscoring the profound disability caused by stroke. Consequently, there is an urgent imperative to identify the factors associated with stroke and its severity [10].

Multiple epidemiological studies have investigated the relationship between serum magnesium levels and the diagnosis of individuals with AIS, producing contradictory results. You et al. discovered an independent association between low magnesium levels and in-hospital mortality among AIS patients [11]. However, other studies reported no significant association [12]. Thus, the rationale of this study is to determine the frequency of hypomagnesemia and its relationship with severity among patients of ischemic stroke presenting to a tertiary care hospital.

## Materials and methods

Study setting and sample collection

A cross-sectional study was carried out at Jinnah Hospital in Lahore, Pakistan, spanning from October 2018 to March 2019. Patients who refused to provide consent for study participation were excluded. Additionally, individuals with a prior history of stroke, pre-existing disabilities, or thyroid disorders identified through history and examination, and patients with chronic kidney disease stage 3 or more and with decompensated liver cirrhosis were also excluded from the study. However, this study included individuals aged between 45 and 90 years who experienced their initial acute episode of ischemic stroke within three days of symptom onset. Following the selection criteria, patients or their relatives were approached to obtain informed consent for participation in the study. A blood sample of around 5 mL was collected through venipuncture, ensuring aseptic techniques were employed, to measure serum magnesium levels accurately. These samples were then sent to the pathology laboratory at Allama Iqbal Medical College in Lahore for detailed analysis. The principle employed was spectrophotometry using a colorimetric and point technique, with the reagents utilized being ammonium caproic acid and Xylidyl blue reagent. The normal range for magnesium levels was 1.8-2.4 mg/dL. During the assessment process, the modified Rankin scale was utilized at the time of admission to evaluate the patient's functional status. The scores obtained from this assessment were meticulously recorded in the Performa, providing a structured and organized approach to data collection and analysis. This method ensured that the patient's clinical status and outcomes could be effectively monitored and evaluated throughout the study.

Sample size

A sample size of 100 was established based on a 95% confidence level and an 8% margin of error.

Ethical consideration

This study obtained approval from the review board's ethics committee of Allama Iqbal Medical College/Jinnah Hospital, Lahore, with reference number 71st/ERB, guaranteeing adherence to the principles outlined in the Declaration of Helsinki. Participants were duly informed, their anonymity safeguarded, and their consent obtained in written form, adhering to ethical standards and protocols.

Statistical analysis

Data were gathered, organized, and statistically analyzed using IBM SPSS Statistics for Windows, Version 27.0 (IBM Corp., Armonk, NY). The continuous variable of age was presented as mean and standard deviation (SD). Categorical data such as gender, hypomagnesemia, and severity of stroke were depicted in terms of frequency and percentage. Stratification was performed based on age, and gender to control effect modifier. Post-stratification involved the application of the chi-square test. Statistical significance was determined as a *P*-value equal to or below 0.05.

## Results

The research involved 100 individuals who had been diagnosed with AIS. The largest proportion of these individuals fell within the 60-74 age bracket, making up 38% of the total sample. The average age of the participants with SD was 65.45 ± 11.8 years. Among the 100 patients, 53 (53%) were male, while 47 (47%) were female. Hypomagnesemia was identified in 35 (35%) patients, with a magnesium level (mean ± SD) of 1.93 ± 0.37 mg/dL upon admission. A comprehensive breakdown of all demographic characteristics of the study subjects can be found in Table 1.

**Table 1:** Demographic attributes of patients. SD, standard deviation

Variables		Frequency (*n*)	Percentage (%)
Age (years) (mean ± SD)		65.45 ± 11.8	
Age group (years)	45-59	35	35
	60-74	38	38
	75-90	27	27
Gender	Male	53	53
	Female	47	47
Severity of stroke	Mild	41	41
	Moderate	32	32
	Severe	27	27
Mg level (mg/dL) (mean ± SD)		1.93 ± 0.37	
Hypomagnesemia	Yes	35	35
	No	65	65

In Table 2 and Table 3, the distribution of stroke severity and hypomagnesemia among males and females and across different age groups show no significant difference, with *P*-values above 0.05. These findings suggest that there is no substantial association between gender or age group and the severity of stroke or the presence of hypomagnesemia among individuals diagnosed with AIS at the tertiary care hospital.

**Table 2:** Distribution of parameters in gender. ^*^Chi-square test.

Parameters		Male	Female	*P*-value*
Severity of stroke	Mild	20	21	0.779
	Moderate	18	14	
	Severe	15	12	
Hypomagnesemia	Yes	17	18	0.287
	No	36	29	

**Table 3:** Distribution of parameters based on age group. ^*^Chi-square test. ^**^Fisher's exact test.

Parameters		Age group (years)			*P*-value*
		45-59	60-74	74-90	
Severity of stroke	Mild	15	13	13	0.406**
	Moderate	8	16	8	
	Severe	12	9	6	
Hypomagnesemia	Yes	9	14	12	0.591
	No	26	24	15	

Table 4 exhibits the prevalence of hypomagnesemia among patients with different severities of stroke. A total of 16 (39%) patients had a mild stroke, 10 (31.3%) had a moderate stroke, and 9 (33.3%) had a severe stroke with hypomagnesemia. A value of *r* = 0.099 indicated a weak positive relationship between the severity of stroke and the presence of hypomagnesemia, suggesting that there is little to no association between the two variables.

**Table 4:** Frequency of hypomagnesemia among patients with different severities of stroke.

Severity of stroke	Hypomagnesemia	Frequency (*n*)	Percentage (%)
Mild	Yes	16	39
	No	25	61
Moderate	Yes	10	31.3
	No	22	68.8
Severe	Yes	9	33.3
	No	18	66.7

## Discussion

Stroke, being the primary contributor to disability and mortality on a global scale, proved to be a burden on developing countries, with over 80% of all strokes occurring in these regions [3]. Research conducted in these areas reveals significantly elevated fatality rates compared to other parts of the world. Magnesium, being the second most abundant intracellular cation, is an indispensable mineral for human health [13] for its crucial role in cardiovascular anti-thrombotic and anti-inflammatory features [14].

In the current study, the average age of patients with SD was 65.45 ± 11.8 years, with a distribution of 53% male and 47% female individuals, aligning with the research by Zeb et al., who reported a mean age (± SD) of 65.5 ± 7.67 years, with 107 (51.94%) male and 99 (48.05%) female patients [15]. Our results were also comparable to those of Ryu et al., where the mean age with SD was 66 ± 12.9 years, predominantly comprising 651 (64.7%) male patients [16]. Ghayyur et al. noted an average age with SD of 60.47 ± 7.78 years, with a male-female ratio of 56.4% to 43.6% [17]. Further, Fatima et al. found a mean age with SD of 60.82 ± 9.30 years, evenly split between 50% of males and females in their ischemic stroke patient cohort [18]. In contrast, Nasim et al. reported a younger mean age with an SD of 51.2 ± 8.4 years, with a higher proportion of patients aged between 41 and 65 years and a male predominance (69.52%) in their study sample, indicating variations in age distribution and gender ratios across different studies [19].

In our study, the majority of patients fell within the 60-74 age range, with 35% exhibiting hypomagnesemia, and magnesium level (mean ± SD) of 1.93±0.37 mg/dL upon admission. Contrasting these findings with prior research by Khan et al., a mean magnesium level of 1.71 mg/dL, with an SD of 0.51 was reported in patients with AIS, showing a hypomagnesemia prevalence of 32% [9]. Similarly, Raju et al. observed standard levels of magnesium of 1.19 mg/dL in cases, considerably smaller than the control group's 2.16 mg/dL (*P *= 0.01), indicating a statistically significant difference [5].

The severity of stroke at admission is a crucial predictive parameter, and studies have shown varying results regarding the relationship between these variables [20]. In this study, the Spearman correlation value of 0.099 suggested a weak positive correlation between the severity of stroke and magnesium levels among individuals experiencing AIS. Contrarily, Khan et al. highlighted a strong negative correlation (*r *= -0.674) between magnesium levels and modified Rankin scale (MRS) scores, indicating a more severe stroke with lower magnesium levels [9].

Further, this study did not identify any links between gender, age, magnesium levels, and disability upon admission. This was supported by Siegler et al. who reported no significant differences in stroke severity based on admission magnesium levels, suggesting that magnesium groups at baseline were not predictive of poor outcomes [21]. Similarly, Bayir et al. did not find significant correlations between neurological deficits and serum magnesium levels in ischemic and hemorrhagic stroke patients in Turkey [12]. In contrast, Adhikari and Gorkhaly investigated magnesium levels in patients with AIS at a tertiary care center in Nepal and found a positive association between levels of magnesium serum and Glasgow Coma Scale (GCS) scores at presentation, indicating that patients with lower magnesium levels tended to have poorer GCS scores. Among stroke patients with hypomagnesemia, a higher proportion had GCS scores of 11/15, whereas those with normal magnesium levels predominantly scored 15/15. However, Pearson correlation analysis between MRS scores and serum magnesium levels in stroke patients revealed a negative correlation of -0.686, -0.734, and -0.880 on days 1, 3, and 7 respectively. This suggests that lower serum magnesium levels in patients with AIS were associated with poorer functional status based on the scores recorded on days 1, 3, and 7 [22].

Limitations

The primary limitation identified is the small sample size, which poses a significant challenge in drawing robust conclusions. Furthermore, the presence of other elements, such as chronic comorbidities, that have not been adequately addressed adds complexity to the analysis. To enhance the correlation and reliability of findings, it is recommended to conduct large multicentric trials so it can help mitigate the impact of small populations and strengthen the validity of the results by encompassing a broader and more diverse participant pool.

## Conclusions

In conclusion, the investigation into the frequency of hypomagnesemia and its correlation with the severity of AIS among patients at a tertiary care hospital sheds light on significant aspects of stroke management. Through meticulous analysis and stratification based on the severity of stroke, this study found a weak positive relationship between the severity of AIS and the presence of hypomagnesemia. However, no statistically significant association was observed between gender or age group and stroke severity or hypomagnesemia. These findings suggest that further research is needed to understand the role of hypomagnesemia in AIS and its potential implications for patient management.
